# Architecture for a Mobile Robotic Camera Positioning System for Photogrammetric Data Acquisition in Hydroelectric Tunnels

**DOI:** 10.3390/s23167079

**Published:** 2023-08-10

**Authors:** Ryan Keizer, Rickey Dubay, Lloyd Waugh, Cody Bradley

**Affiliations:** 1 Department of Mechanical Engineering, University of New Brunswick, Fredericton, NB E3B 5A3, Canada; dubayr@unb.ca; 2 Department of Civil Engineering, University of New Brunswick, Fredericton, NB E3B 5A3, Canada; waugh@unb.ca; 3 Bradley Engineering Ltd., Estey’s Bridge, NB E3G 6M7, Canada; cody@bradleyengineering.ca

**Keywords:** photogrammetry, hydroelectric infrastructure, robotic systems, infrastructure inspection, advanced controls

## Abstract

The structural condition of hydroelectric tunnels is important to the overall performance, safety, and longevity of generating stations. Significant effort is required to inspect, monitor, and maintain these tunnels. Photogrammetry is an effective method of collecting highly accurate visual and spatial data. However, it also presents the complex challenge of positioning a camera at thousands of difficult-to-reach locations throughout the large and varying-diameter tunnels. A semi-automated robotic camera positioning system was developed to enhance the collection of images within hydroelectric tunnels for photogrammetric inspections. A continuous spiral image network was developed to optimize the collection speed within the bounds of photography and capture-in-motion constraints. The positioning system and image network optimization reduce the time and effort required while providing the ability to adapt to different and varying tunnel diameters. To demonstrate, over 28,000 images were captured at a ground sampling distance of 0.4 mm in the 822 m long concrete-lined section of the Grand Falls Generating Station intake tunnel.

## 1. Introduction

High-resolution visual and spatial models are enhancing traditional methods of inspecting large-scale structures. Data collection for these models can be accomplished with lidar; however, photogrammetry can produce very high-resolution RGB point clouds at high speeds that can be inspected visually. These improved models overcome the inaccuracy and uncertainty of traditional inspection methods and enable a better understanding of infrastructure condition and deterioration trends.

**Figure 1:**
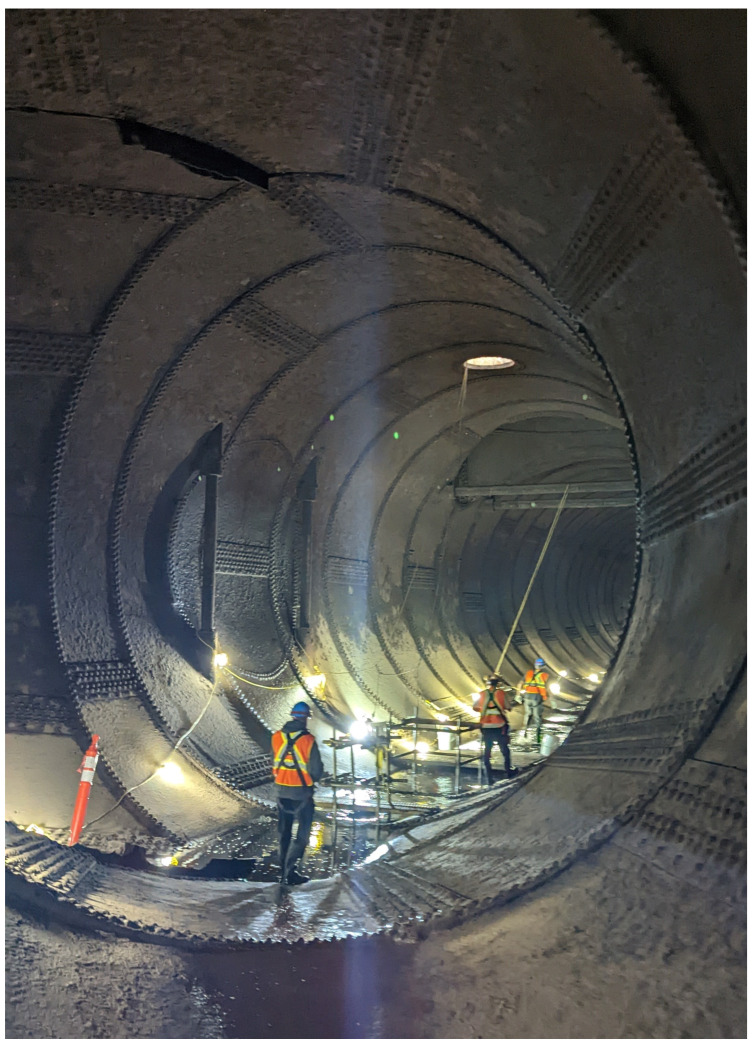
Grand Falls intake tunnel, steel-lined distribution section.

Typical hydroelectric generating stations have multiple penstocks and often include additional associated tunnels. These tunnels are often kilometers long and upwards of 10 m in diameter and beyond; the Grand Falls Generating Station (Grand Falls) intake tunnel is 7.5 m in diameter, and the concrete-lined section is 822 m long, for example. The current methods of collecting photogrammetric data at the quality and resolution desired for inspection consist primarily of manual tasks that demand great labor efforts, especially in large tunnels. This process is time intensive and takes longer than a traditional walk-through inspection by an inspector. With individual generating stations having different diameter tunnels or multiple different stations being inspected by the same equipment, tunnel-specific image capture methods are inefficient, costly and require substantial modifications to capture unique locations. Figure 1 shows the distribution section of the Grand Falls intake tunnel, providing an example of the size and conditions of these tunnels.

Station shutdowns come at a significant expense as replacement electricity must be outsourced during the downtime. The Mactaquac Generating Station (Mactaquac), a generating station near Grand Falls, has a 670 MW capacity, whereas Grand Falls has a capacity of 66 MW. For example, the cost of replacement electricity for the smaller Grand Falls station was estimated to be $38,000 per day during the 2017 shutdown, which lasted for 10 days in 2017 [1]. The cost increases for larger generating stations and can be substantially higher for unplanned shutdowns. The image collection process consumes a significant portion of these downtimes, signifying how impactful the data collection speeds are to the overall maintenance expense.

Identifying these limitations and challenges in the process led to the understanding that introducing robotic and automated elements to the image acquisition process could significantly increase data acquisition speed and also allow a wide range of tunnels to be inspected with a single system while maintaining consistent model quality and properties. This evolved into us asking the following question: What data acquisition system design would be best suited to improve the efficiency of the photogrammetric data collection process in a tunnel, while accounting for tunnel diameter variability and transportability?

The goal of this research was to develop a mobile robotic camera positioning system that improves the efficiency of the tunnel image collection process while prioritizing adaptability and transportability through a variable and modular design. The system is referred to as the Automated Tunnel Inspector (ATI). The term automated in this context refers to the system being operated by the combined electromechanical and control systems to reduce the effort by humans, based on the Cambridge definition in [2].

The key objectives of this research are as follows:

- To identify and evaluate the optimal image capture sequence to minimize the time required to complete the collection process while maintaining consistent and sufficient image overlap and density.
- To develop a semi-automated robotic image collection system capable of adapting to a range of tunnel diameters and allowing for easy independent transportation into and out of the confined tunnels.
- To collect a complete set of images for the photogrammetric modeling of the Grand Falls Generating Station intake tunnel.
- To evaluate and demonstrate the use of advanced control techniques in unique environments and applications.

Maintaining optimal and consistent image properties is critical for ensuring that the subsequent photogrammetric model is effective. This sets strict constraints on where the images must be captured. Therefore, optimizing the sequence and method whereby these image locations are navigated significantly influences the time required to move throughout a complete network of images.

Transportability in the context of this work pertains to the limited access presented by these tunnels, including small-sized access points within a close-quarter environment and the manual ferrying of the system in and out of the tunnel. In the case of the Grand Falls intake tunnel, access is via a ladder through a small porthole located on the top of a distribution tunnel, which is just large enough for a person to squeeze through. The path to get to this access point involves multiple levels of stairs, tight corridors, and low ceilings. Equipment can be lowered through a separate 0.7 m porthole at the top of the intake tunnel. Addressing this goal includes a design with the ability to be reduced into compact modular components capable of being transported manually by operators into the limited-access tunnels. The adaptability that is required stems from the variations that are present among the large number of hydroelectric tunnels currently in use. Not only are the diameters of the different tunnels unique, but some individual tunnels also contain varying diameters and profiles to be followed. Hence, adapting to these tunnel variations was a key aspect of the research objectives.

These hydroelectric tunnels pose a unique set of constraints and require high performing controls to effectively operate in these conditions. The implementation and evaluation of various control methods in this environment demonstrates the versatility and robustness of these control techniques in harsh dynamic environments.

## 2. Background

### 2.1. Bradley Engineering Ltd. History

The Grand Falls Generating Station (Grand Falls) in New Brunswick, owned by New Brunswick Power Corporation (NB Power), has been in operation for nearly a century and has a total output capacity of 66 MW. The intake tunnel for this station diverts water from the Saint John River and is approximately 921 m long, which includes an 822 m concrete tunnel as well as the manifolds and additional steel lined sections. Traditionally, the inspection of this tunnel has been completed visually with an inspector viewing from the tunnel invert with observations documented by location (longitudinal and clock location), size, and inspector comments on deterioration. Bradley [1,3] identified major limitations in this process; the difficulty of visualizing deterioration trends throughout the tunnel, the low accuracy of defect descriptions, the low accuracy of the defect locations, and the overall subjectivity of the documentation due to differing interpretations by multiple inspectors.

**Figure 2:**
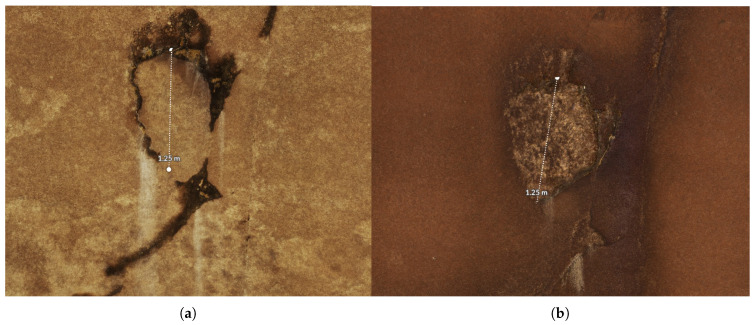
Defect example showing change in area from 2017 to 2022. (**a**) Patched area (2017). (**b**) Hole defect growth from lost patch (2022).

Bradley developed a photogrammetric inspection system capable of capturing high-resolution visual imagery to be processed into 3D models with high spatial accuracy. These data and the resulting models are used to analyze defects such as cracks and dislocated concrete; Figure 2 shows an example of a concrete patch that was washed out between 2017 and 2022.

Capture of these images requires positioning a camera in difficult-to-reach places throughout the tunnel. To aid in positioning the camera, the Tunnel Inspection Assistant (TIA) was designed specifically for the Grand Falls tunnel and incorporated associated photogrammetric features. It consisted of a three-wheeled triangular frame and a boom used to rotate the camera a set radius around the center of the tunnel. The TIA was manually maneuvered with one steerable wheel. The rotating boom was motor driven and pre-programmed to follow a set incremental routine. The process of scanning the tunnel involved positioning the TIA in the center of the tunnel, checking for level and alignment, capturing images through a planar rotation perpendicular to the tunnel axis, and incrementing along the tunnel a set distance to the next station to repeat the process. The initial capture of the 822 m concrete-lined section of Grand Falls took a total of seven days to complete. The result of the process was a 3D model of the tunnel with a spatial accuracy of approximately 5 mm and a ground sampling distance of 1.3 mm.

The resulting model from Grand Falls demonstrated to NB Power the concept and effectiveness of this photogrammetric inspection system. NB Power had also been monitoring Mactaquac with traditional methods. This station suffers from concrete expansion in the dam due to an alkali aggregate reaction. The measurement precision required to assess the condition of this effect makes Mactaquac an opportune area to further assess the abilities of the photogrammetric inspection system.

However, TIA was a one-off design made specifically for assisting with the image capture of the Grand Falls intake tunnel, and therefore, a modified version of the TIA was developed for the penstocks in Mactaquac [4]. Throughout this time, the inspection process developed by Bradley et al. [1,3,4] fueled the launch of Bradley Engineering (BE) in 2018, a company that conducts photogrammetric operations. The extensive experience acquired by BE in performing these operations has led to the identification of additional opportunities for improvement in the previous image capture process. As experienced when moving from the Grand Falls tunnel to the Mactaquac penstock, the customized design for a specific tunnel’s dimensions hinders the efficiency of the capture system. The manual efforts required to transport and utilize the TIA systems limit the speed of the data acquisition process. Study of this work led to the identification of the opportunity for a multipurpose automated data acquisition system that can adapt to various tunnel dimensions and configurations.

### 2.2. Inspection Technologies

There are many types of inspections; one type is a visual inspection that can encapsulate a broad range of methods and technologies. With advancing photographic technology and image processing techniques, visual inspections are an opportune area for advancements in automation. The following subsections examine some of the current technologies and methods used to automate portions of the visual inspection process. After reviewing the factors that influence researchers to explore automation in the inspection field, it is apparent that the data acquisition process is the primary phase that demands automation.

#### 2.2.1. Unoccupied Underwater Vehicles

Testing began on underwater robots several decades ago [5]. These robots avoid the need for draining a tunnel and are currently useful for identifying significant structural movement, as noted in the exploration of utilizing an underwater ROV in the monitoring of a hydroelectric tunnel in [6,7]. This was used in combination with a range of acoustic sensors for identifying potential deformations, cracks, or debris within the tunnel, which could then be further analyzed during the next de-watered manual inspection. While this demonstrates the effective preliminary identification of potential areas of concern through spatial monitoring, the visual inspection and documentation is still conducted manually by an inspector during shutdowns. The quality of these relatively low resolution images collected underwater is significantly vulnerable to the underwater conditions [8], although recent studies have demonstrated effective photogrammetric results in various environments [9].

#### 2.2.2. Unoccupied Aerial Vehicles

Due to increased constraints and complexities, the autonomous indoor use of UAVs has been limited. However, work from Özaslan et al. [10,11,12] has progressed towards automating the image capture process for visual inspections of penstocks and tunnels with a micro aerial vehicle (MAV) with the goal of collecting stitched 360-degree panoramic images from incremental sections along the tunnel for 3D reconstruction. The MAV system was based around a hex-rotor platform with four cameras directed evenly around the cross section of the tunnel and an onboard LED arrangement to provide lighting. While utilizing a complete onboard lighting system helps to ensure consistent and sufficient lighting throughout the process, this does increase the space and weight requirements, which can be a limiting factor in flight capabilities. With a loaded flight time of 10–15 min, the proposed system is limited in the size of infrastructure that can be captured quickly, a common drawback of UAV systems. The underlying limitation of this type of system is the aerial vehicle’s load capacity; this hinders the flight times and the ability to utilize larger, higher-quality cameras and lighting systems that are necessary to produce high-resolution 3D models.

An important aspect to consider when selecting a vehicle type for a specific application is the amount of control required. In general, UAVs have more agility and maneuverability than terrestrial vehicles. However, the additional degrees of freedom (DOF) associated with UAVs brings further complications to the positioning and navigation of these vehicles [10].

#### 2.2.3. Terrestrial Vehicles

Ground-based or terrestrial vehicles are generally more familiar and have been adapted to a larger range of applications than UAVs, making them an obvious option to implement automation in the inspection process. Comparatively, terrestrial vehicles have increased load capacity and a reduced complexity of maintaining stability and control under loads; however, the terrain being traversed is an additional element not faced by UAVs. This aspect was a limiting factor in the work carried out by Hosotani and Yamamoto [13], who attempted to create a self-propelled image capturing robot to reduce effort in the inspection of free flow tunnels. The goal was to capture panoramic images of the wall surface from the center of the tunnel with a hemispherical camera on a two-wheel drive platform with an ultrasonic sensor monitoring its distance from the wall. After testing in a 100 m tunnel, the images were unsatisfactory for current inspection standards, as distortions and inconsistencies were present due to the meandering and instability of the robot on the rough tunnel floor surface.

With the comparable goal of collecting high-quality images of a tunnel, Stent et al. [14] explored a more stable and rigid approach to solving this task with two DSLR cameras that rotate around the center of the tunnel. The capture system was mounted to a monorail that runs the extent of the tunnel, which significantly limits the areas in which it can be utilized and is not easily transferable to other tunnels. The high level of detail produced allows for sub-0.3 mm cracks to be identified, which is a resolution that is not attained by many systems in this environment.

A visual inspection for crack detection in concrete structures was trialed by Yu et al. [15] based on a small mobile robot. A similar mobile robot was used [16,17] that included an array of ultrasonic sensors to detect deformations in tunnels. Though experiments of these systems were performed in multiple environments (indoor, road and subway tunnels) [15,16,17], they all had generally flat floor surfaces. The small frames of these mobile robots are likely unable to effectively move through the curved, rough and sloped terrain of many hydroelectric tunnels, and it does not appear feasible to transport them into a hydro-electric tunnel.

A crawler teleoperation robot system was implemented in [18] to perform a range of image and video collections within diversion tunnels. While the inspection covered the entirety of the tunnel surfaces, data acquisition occurred at 5 m intervals, where each interval took 5 h to complete; in tunnels potentially kilometers long, where downtime is expensive, this rate of acquisition is not feasible for many dam applications. A crawler-type robot was also the foundation of the autonomous inspection system developed by Kamiyama [19], in which construction site embankments were inspected. The crawler robot was able to climb slopes of 45 degrees and surmount walls upwards of 450 mm, demonstrating the capabilities and maneuverability benefits of crawler technology for this application. The slow collection speeds that the crawler system provides is not feasible for scanning the entirety of a tunnel such as Grand Falls in the time frame of a single shutdown.

## 3. Theory and Methodology

This section details the general methodology and provides a theoretical background on some key aspects of the analysis process.

### 3.1. Serial Manipulator

Serial manipulators are defined as a set of links and joints connected in series that provide relative motion of adjacent links. This type of manipulator applies to a significant portion of the system outlined in this project.

#### 3.1.1. Spatial Relation

The motion of *n*-DOF serial manipulators were studied extensively for adaptation into the system selected for manipulating the camera position. The spatial description of the system can be defined using the homogeneous transformation matrices $\left({}_{n+1}^{n}T\right)$, as shown in Equation (1). This defines the position and orientation of successive joints based on the mDH convention, as defined in [20]. Variables $x_{n},y_{n},$ and $z_{n}$ are unit vectors describing the frame axes of joint *n* and are combined to create the rotation matrix expressing frame *n*+1 to frame *n*. Additionally, ${}^{n}p_{n+1_{orig}}$ depicts the location of the origin of *n*+1 relative to *n*, broken down into vectors ${}^{n}p_{i}$.

(1) $${}_{n+1}^{n}T=\left[\begin{array}{cccc} x_{n+1}\cdot x_{n} & y_{n+1}\cdot x_{n} & z_{n+1}\cdot x_{n} & {}^{n}p_{x}\\ x_{n+1}\cdot y_{n} & y_{n+1}\cdot y_{n} & z_{n+1}\cdot y_{n} & {}^{n}p_{y}\\ x_{n+1}\cdot z_{n} & y_{n+1}\cdot z_{n} & z_{n+1}\cdot z_{n} & {}^{n}p_{z}\\ 0 & 0 & 0 & 1 \end{array}\right]=\left[\begin{array}{cccc} \; & \; & & \\ \; & \left({}_{n+1}^{n}R\right) & \; & {}^{n}p_{n+1_{orig}}\\ \; & \; & & \\ 0 & 0 & 0 & 1 \end{array}\right]$$

Compounding these transformations through *n* joints relates the pose of the camera on the ATI to the base as follows in Equation (2).

(2) $$T_{cam}^{base}={}_{1}^{base}T*{}_{2}^{1}T*\ldots *{}_{n}^{n-1}T*{}_{cam}^{n}T\;$$

#### 3.1.2. Kinematics and Dynamics

The outlined spatial description is used in the derivation of the Jacobian matrix (*J*, 6 x *n*) of the manipulator, which defines the relationship between the camera velocity (ν, 6 x 1) and the joint velocities ($\dot{\Theta }$, *n* x 1) as follows (3):

(3) $$\nu =J*\dot{\Theta }\;$$

Incorporating the mass and inertia of the system members, along with the frictional effects on moving joints and surfaces, the torques and forces can be evaluated. By defining the desired motion profiles of the driving joints, the dynamic responses of the joints can be analyzed throughout the defined trajectory. The general dynamic equations for an *n*-DOF manipulator, as derived from the Newton–Euler equations [20], are represented in Equation (4). MapleSim^™^ is used to demonstrate the 3D motion and analyze the dynamic response more thoroughly, as demonstrated in Section 4.

(4) $$\tau =M\left(\Theta \right)\ddot{\Theta }+V\left(\Theta ,\dot{\Theta }\right)+G\left(\Theta \right)+F\left(\Theta ,\dot{\Theta }\right)\;$$

Here, τ is the *n* x 1 vector of joint forces/torques, M(Θ) is the *n* x *n* mass/inertia matrix, $V(\Theta ,\dot{\Theta })$ is an *n* x 1 vector collecting centrifugal and Coriolis terms, G(Θ) is an *n* x 1 vector of gravity terms, and $F(\Theta ,\dot{\Theta })$ is the inclusion of viscous and Coulomb friction effects. Θ depicts the position of each joint, with $\dot{\Theta }$ and $\ddot{\Theta }$ being the first and second derivatives, respectively.

### 3.2. Vehicle Dynamics Theory

The mobile robotic system that is being explored in this project may not resemble a typical road vehicle, but the generic electro-mechanical principles remain applicable. This section presents the theories behind the fundamental operation and stability of dynamic vehicles.

The system discussed within this project is not a traditional vehicle, being limited in speed, application, and complexity; a simplified, one-dimensional description of the vehicle dynamics is discussed and applied for this portion of the work. The vehicle’s motion is understood by analyzing the power and forces exerted against the external forces applied onto the vehicle, which is dynamically modeled as shown in Equation (5).

(5) $$M_{v}\frac{dV}{dt}=\sum F_{Tractive}-\sum F_{Resistive}=\left(F_{tf}-F_{tr}\right)-\left(F_{rf}+F_{rr}+F_{D}+F_{g}\right)\;$$

The element $M_{v}$ is the total mass of the vehicle, $\frac{dV}{dt}$ is the linear acceleration, and $F_{Tractive}$ is the net tractive effort provided by the wheels of the vehicle, which includes the efforts of both the front ($F_{tf}$) and rear ($F_{tr}$) wheels. The total resistive force ($F_{Resistive}$) experienced by the vehicle incorporates the rolling resistance of each wheel ($F_{rf}$) and ($F_{rr}$), aerodynamic drag resistance ($F_{D}$), and grade resistance ($F_{g}$) [21]. As the system developed in this project operates in a confined indoor space at very low speeds and with a skeletal structure, the effect of aerodynamic drag can be ignored for this analysis.

Rolling resistance is a factor of the ground reaction force causing a resistive force on the rotation of the tires due to the deflection of the tire. It varies depending on the type of tire and material, tire pressure, and type of surface being driven on. The tire deflection causes the ground force to shift towards the leading edge of the tire, imposing an eccentric load on the tire and therefore creating a counter moment on the tire. When moving along sloped surfaces, the mass of the vehicle imposes a force acting downward in correlation with the force of gravity. While ascending a slope, this acts against the vehicle motion, referred to as grade resistance, whereas it acts as a supporting force on motion while descending a slope; however, for analyzing the functional ability and performance of a vehicle, this aspect will be neglected as this scenario demands significantly less effort from the driving system as compared to the upward slope case [21].

The above parameters outline the minimum tractive effort required to properly operate the vehicle and overcome the resistive efforts. Additionally, the upper limit of the tractive efforts is the maximum tractive effort that can be applied while maintaining traction and avoiding slippage. Any additional force beyond this limit will result in the tire beginning to slip on the surface [21].

Additional details on this theory and how it has been utilized in this system is discussed in [22].

### 3.3. Controls Theory

Model predictive control (MPC) makes model-derived predictions of the system response to better adjust the control action (*u*) for current and future time steps. It does this based on the concept of minimizing an objective cost function (*J*), which primarily includes the tracking of the response error [23,24]. Additional elements may be added into the cost function to consider the variation of the nominal control variables and violation of applied constraints [23,25].

The MPC architecture works on the premise of the creation of a dynamic matrix (*A*), with dimensions *P* x $n_{u}$, as can be seen in Equation (6), equivalent to the prediction horizon (*P*) and the control horizon ($n_{u}$). The prediction horizon is determined through collecting the open-loop response from the system model reacting to a step input and is the number of discrete time steps required for the response to reach within 95% of the steady state value. Analytical approaches can also be used. The dynamic matrix is then filled with the corresponding open-loop response values ($a_{i}$) at discrete time steps up to the prediction horizon. The control horizon represents the number of future control actions being considered in the evaluation [23,26].

(6) $$A=\left[\begin{array}{cccc} a_{1} & 0 & \cdots & 0\\ a_{2} & a_{1} & \cdots & 0\\ \vdots & \vdots & \ddots & \vdots \\ a_{P} & a_{P-1} & \cdots & a_{P-n_{u}} \end{array}\right]$$

The cost function in Equation (7) represents the function used to optimize the control action, including the addition of a move suppression factor λ applied to the control action used to ensure a non-singular solution.

(7) $$J=\sum_{i=1}^{P}{\left({\hat{Y}}_{t+i}-R_{t+i}\right)}^{2}+\sum_{i=1}^{n_{u}}\lambda \left(\Delta U_{t+i-1}\right)\;$$

The variable ${\hat{Y}}_{t+i}$ is the prediction vector produced from the system model and dynamic matrix, $R_{t+1}$ is the reference trajectory, both being considered *i* time steps ahead of the current time step (*t*). The least squared method is used to optimize the cost function and determine the control action, as shown in Equation (8), with the current predicted error vector ($\vec{E}$) depicting the current discrepancy from the reference trajectory vector ($\vec{R}$).

(8) $$\Delta U={\left(A^{T}A+\lambda I\right)}^{-1}A^{T}E$$

The MPC provides a robust feedback control technique for controlling SISO and MIMO systems that are highly coupled and subject to the unique constraints of the systems. Extensive details on MPC can be found in [25,26], and additional details on the application of MPC in this system are in [22].

## 4. Detailed Design and Analysis

The design that is detailed within this is the result of several conceptual iterations. Many potential designs were conceptualized, compared and evaluated before selecting the ATI design discussed here. These were fundamentally evaluated based on their anticipated ability to complete the required camera maneuvers and their level of adaptability. To be concise and focused, the details of this thorough evaluation process are excluded from this paper; however, extensive discussions are provided in [22,27].

The ATI system integrates a total of sixteen linear or rotational actuations to perform nine unique actions that achieve the camera articulation and global navigation needed to collect the required images. The entire vehicle system and all of its actuators are powered through a single battery source, while the camera systems have their own separate batteries. Connecting the sixteen different actuators requires the substantial management of the cables and connections, including shielding the cables, to avoid noise and convoluted tubing for protection from the environment. The following sections detail the required kinematics and dynamics as determined through simulation and calculations, the actuators and setup used to complete the actions, and the rationale for these conclusions.

A combination of robotic kinematic theory, general mechanical engineering principles, and dynamic simulation software (MapleSim^™^ [28]) laid the foundation for understanding the kinematic and dynamic demands of each ATI joint. Simulations were used at various stages of the design and analysis process, initially to determine and visualize the kinematics of each joint and driving actuator throughout the entire range of motion (ROM) of the cameras. This subsequently provided the means for the dynamics of each driving member to be evaluated through that same range, providing information and specification targets for component selection. The ATI can be divided into three independent sections for these evaluations and simulations; the arm, the boom, and the ATI base.

### 4.1. Arm Manipulation

The manipulation of the arm accounts for the radial adjustment of the arm length, the orientation of the cameras, and the rotation of the complete arm. These actuations occur after the arm as a whole has been positioned and aligned with the center axis of the tunnel. The orientation of the camera equipment is managed by two rotary stage actuators at the end of each arm. Completing the radial adjustment of the ATI arm is achieved with two synchronized linear actuators driving telescopic members that hold the orientation actuators and photography equipment. The entire arm is mounted on a stepper motor-driven shaft at the tip of the boom; the anticipated operational rotation speed for most applications is in the range of 1–3 degrees per second and can move quicker in non-capture scenarios.

**Figure 3:**
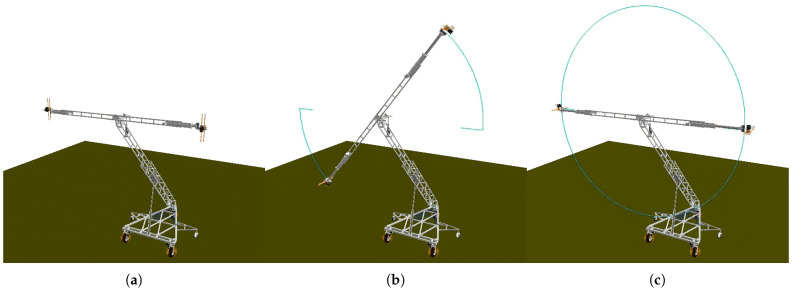
Simulation of arm manipulation components: orientation rotation, arm extension, arm rotation. (**a**) Start position (t = 0 s). (**b**) Mid-operation (t = 17 s). (**c**) End position (t = 30 s).

For this subsystem of the ATI, the desired motion of each joint is defined and therefore acts as the direct input for the dynamic analysis conducted. For the dynamic simulation, the orientation change drove the rotary joint through a 90${}^{\circ }$ rotation, with each arm extended radially by 0.45 m, and then rotated and stopped after 180${}^{\circ }$. The simulation spanned 30 s, with each actuation being performed sequentially. The actions performed within the simulation were set at higher velocities and accelerations (1.5–3 times higher) than were anticipated to be applied in practice, providing conservative information and allowing for flexibility in future applications. Figure 3 visualizes the ATI at the start, end, and an intermediate position of the simulation, with a tracing of the camera position throughout.

**Figure 4:**
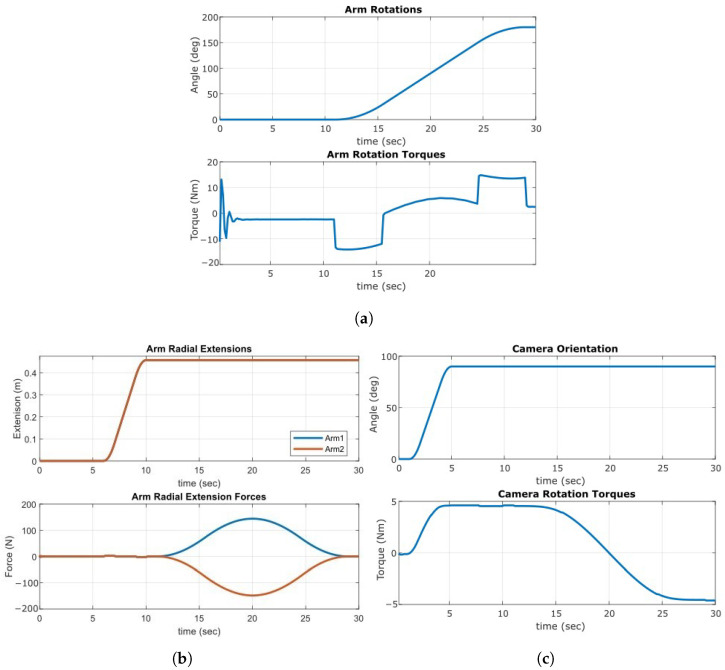
Dynamic responses of arm system components. (**a**) Arm Rotation. (**b**) Arm Extension. (**c**) Camera Orientation.

The arm was simulated through a rotation of 180 degrees to and from a stationary position over 18 s, resulting in a peak rotational speed of 13.3 degrees per second. This operation is performed with a geared stepper motor providing a nominal output torque of 110 Nm up to a speed of 4 rpm (24 degrees per second). Figure 4a shows the torque response of arm rotation throughout the defined motion profile. Figure 4b shows the dynamic response of the radial extension joint. The actuators used provide a 0.45 m stroke with 890 N of dynamic force. Optical feedback encoders on each actuator are used to match both the speed and position throughout the trajectory. Section 5.1 details the control techniques for this action. A 4 s orientation toggle was simulated with a peak speed of 30 degrees per second; this is two times faster than the rated speed of the rotary actuator used. Figure 4c shows the torque response of this actuator during the change in orientation and throughout the other arm-manipulating actions.

### 4.2. Boom Manipulation

The centering and alignment of the entire arm is manipulated through the planar boom system consisting of three joint actuations: boom extension, boom inclination, and the tip inclination. The position of the boom tip relative to the base is altered with the extension and inclination of the boom. The rotation of the boom tip joint adjusts the pose of the arm center frame, allowing for its alignment with the tunnel axis to be maintained.

#### 4.2.1. Simulation Analysis

**Figure 5:**
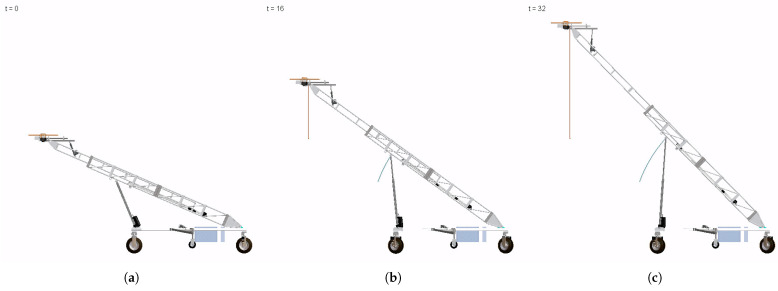
Simulation of boom manipulation components: boom extension, boom inclination, tip inclination. (**a**) Starting low position (t = 0 s). (**b**) Mid-operation (t = 16 s). (**c**) End elevated position (t = 32 s).

The simulation performed on this section of the ATI demonstrates the manipulation of the ATI arm frame throughout a range of typical operating positions and to the extremes of its ROM, as shown in Figure 5. A consistent frontal offset of 2 m is maintained between the base and the rotation plane of the camera arm throughout all positions. A pure vertical translation of the arm frame is completed from a fully lowered position, with the arm center elevated to 2.25 m (Figure 5a) to full extension and with the arm center at 4.5 m from the tunnel invert (Figure 5c). This range demonstrates the capabilities of the ATI to reach the center and align with the center axis of tunnels with diameters ranging from 4.5 m to 9 m.

**Figure 6:**
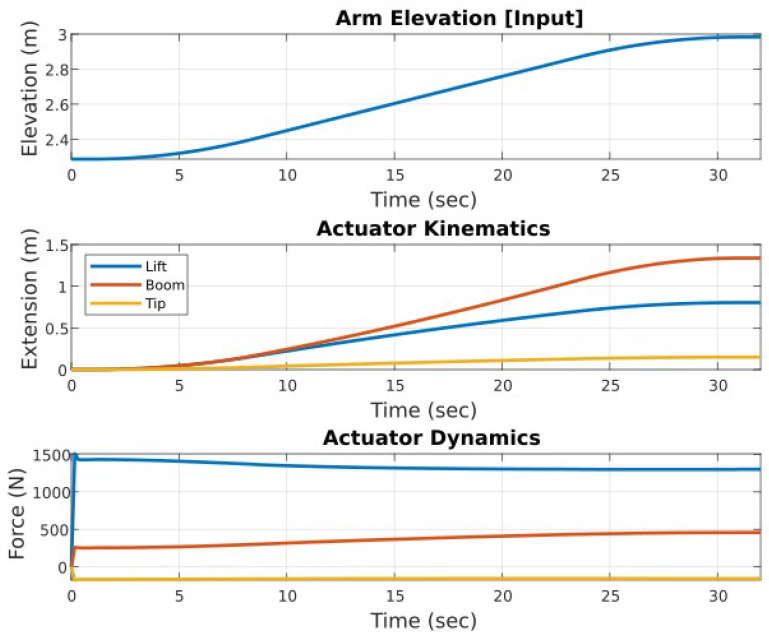
Arm frame elevation simulation: inverse kinematics and dynamics.

Unlike the manipulation of the arm and camera components, the elevation and frontal offset of the arm are coupled and not directly controlled through single independent actuations. An inverse kinematic analysis first determines the motion requirement of each joint and corresponding driving actuators to define the position and trajectory of the arm frame. Subsequently, the calculated motion profiles become the input for a dynamic analysis to determine the corresponding forces and torques to produce the required accelerations to achieve the defined motions. The plot in Figure 6 displays the results from these analyses for the trajectory defined above. Key parameters and extracted values are summarized in Table 1. The simulation of this provides visual and numerical information on critical positions and peak load conditions, and aids the selection and sizing of the driving components. A summary of the key dynamic simulation results is provided in Table 1.

#### 4.2.2. Analytical Analysis

**Figure 7:**
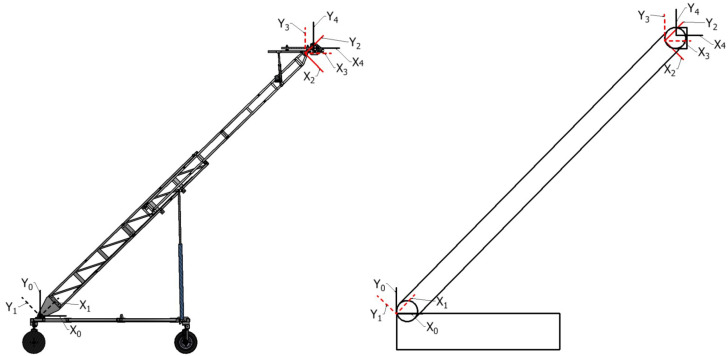
ATI joint frame and mDH parameter definitions.

The generalization of the kinematic relationship between each driving joint and the end arm position is defined through the analytical analysis, which is derived from both the simulated model and the modified Denavit–Hartenberg (mDH) conventions. The general kinematic relationship derivation that follows is associated with the spatial description of the ATI boom presented in Figure 7. The parameters defining the relationship of adjacent links for this system are presented in Table 2, following the mDH convention [6]. This description defines the joints that form the Revolute–Prismatic–Revolute planar mechanism of the boom.

The parameters in Table 2 are represented in Figure 7 and are used to formulate the homogeneous transformation matrices between each successive link and are further combined to relate the arm center frame to the reference frame of the base, as defined in Equation (9). This details the forward kinematic relationship between the arm center and the base and is the basis for inversely determining the required joint positions for a defined elevation and frontal offset. This relationship can be expanded from the arm center to one of the end camera positions; however, this does not provide information relevant to this application. A more exhaustive collection of transformation matrices, joint relationships, and derivations within the process are documented in [22]. Notations *c* and *s* (with corresponding subscripts B (boom) and T (tip)) represent the cosine and sine values of $\theta_{i}$, respectively.

(9) $${}_{Arm}^{Base}T=\left[\begin{array}{cccc} c_{B}c_{T}-s_{B}s_{T} & s_{B}c_{T}-c_{B}s_{T} & 0 & l_{2}c_{B}+d_{B}s_{B}+l_{4}\\ c_{B}s_{T}-s_{B}c_{T} & c_{B}c_{T}-s_{B}s_{T} & 0 & l_{2}s_{B}-d_{B}c_{B}+d_{4}\\ 0 & 0 & 1 & 0\\ 0 & 0 & 0 & 1 \end{array}\right]$$

The angle of the boom and the tip joints as well as the length between these joints as altered by the boom length are derived from the inverse displacement analysis. Equations (10) and (11) define these relationships, where $\theta_{B}$ and $\theta_{T}$ are the angle of the boom and tip, respectively, $x_{Tip}$ and $y_{Tip}$ depict the horizontal and vertical position of the tip frame relative to the base origin, and $x_{Arm}$ and $y_{Arm}$ represent the same for the arm frame. Parameter $d_{B}$ defines the stroke of the boom extension actuator, with ${\hat{d}}_{B}$ representing the boom length in the fully retracted position.

(10) $$\theta_{B}=-\theta_{T}=atan2\left(y_{Tip},x_{Tip}\right)=atan2\left(\left(y_{Arm-d_{4}}\right),\left(x_{Arm}-l_{4}\right)\right)$$

(11) $$D_{B}=d_{B}+{\hat{d}}_{B}=\sqrt{x_{Tip}^{2}+y_{Tip}^{2}}=\sqrt{{\left(x_{Arm}-l_{4}\right)}^{2}+{\left(y_{Arm}-d_{4}\right)}^{2}}$$

**Figure 8:**
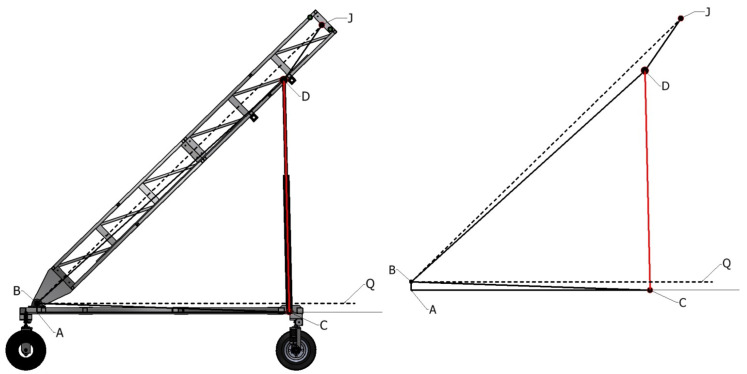
Defining parameters relating driving actuator of boom inclination joint.

**Figure 9:**
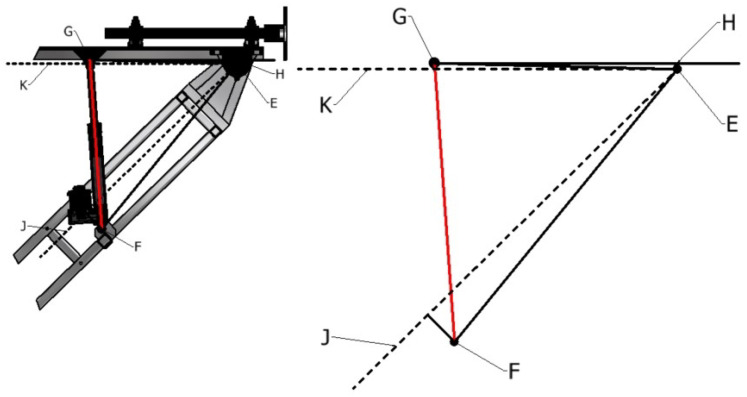
Defining parameters relating driving actuator of tip inclination joint.

Expanding this relationship to incorporate the linear actuator configurations that drive the revolute joints includes the spatial representations defined in Figure 8 and Figure 9. The forward and inverse displacement relationships between the driving actuator length and the resultant angle are defined in Equations (12)–(15) for the boom and tip inclinations, respectively. These relationships are used to control the actuators through the user inputs of tunnel diameter and required frontal offset, which translate to a pose of the arm center relative to the base through known dimensions of the ATI base. Parameters $d_{T}$ and $d_{L}$ define the stroke of the tip and lift actuators, with ${\hat{d}}_{T}$ and ${\hat{d}}_{L}$ representing the respective actuator lengths in the fully retracted positions. Other parameters correspond to those defined in Figure 8 and Figure 9 and are summarized in Table 3.

(12) $$\theta_{B}=acos\left(\frac{-{\left(d_{L}+{\hat{d}}_{L}\right)}^{2}+\alpha_{L}^{2}+\beta_{L}^{2}}{2\alpha_{L}\beta_{L}}\right)+acos\left(\frac{\beta_{L}^{2}-\lambda_{L}^{2}+\rho_{L}^{2}}{2\beta_{L}\rho_{L}}\right)-atan\left(\frac{\gamma_{L}}{\phi_{L}}\right)$$

(13) $$\begin{array}{cc} d_{L} & =\sqrt{-2\alpha_{L}\beta_{L}A_{L}+{\alpha_{L}}^{2}+{\beta_{L}}^{2}}-{\hat{d}}_{L},\\ \mathrm{where},\;A_{L} & =cos\left[\theta_{L}-acos\left(\frac{\beta_{L}^{2}-\lambda_{L}^{2}+\rho_{L}^{2}}{2\beta_{L}\rho_{L}}\right)+atan\left(\frac{\gamma_{L}}{\phi_{L}}\right)\right] \end{array}$$

(14) $$\theta_{T}=acos\left(\frac{-{\left(d_{T}+{\hat{d}}_{T}\right)}^{2}+{\alpha_{T}}^{2}+{\beta_{T}}^{2}}{2\alpha_{T}\beta_{T}}\right)-acos\left(\frac{{\beta_{T}}^{2}-{\lambda_{T}}^{2}+{\rho_{T}}^{2}}{2\beta_{T}\rho_{T}}\right)-atan\left(\frac{\gamma_{T}}{\phi_{T}}\right)$$

(15) $$\begin{array}{cc} d_{T} & =\sqrt{-2\alpha_{T}\beta_{T}A_{T}+{\alpha_{T}}^{2}+{\beta_{T}}^{2}}-{\hat{d}}_{T},\\ \mathrm{where},\;A_{T} & =cos\left[\theta_{T}+acos\left(\frac{\beta_{T}^{2}-\lambda_{T}^{2}+\rho_{T}^{2}}{2\beta_{T}\rho_{T}}\right)+atan\left(\frac{\gamma_{T}}{\phi_{T}}\right)\right] \end{array}$$

#### 4.2.3. Specifications and Components

The inclination of the boom is the largest actuation of the system. Moving the large frame of the boom with the extended arm creates a substantial moment about this joint. Driving this joint with a linear actuator offers a simple way to provide sufficient torque to rotate this joint that a common direct rotary actuator could not supply. The ROM of the boom angle requires a 0.75 m displacement of the driving actuator and a driving force of 1500 N. An actuator providing this stroke with 5000 N of driving force was used, with hall effect sensors providing the high-resolution positional feedback of this actuation.

The inclination of the tip joint closely resembles the operation of the boom inclination with similar configurations. Although the ROM of the rotation are alike, the driving actuator being positioned close to the joint significantly reduces the stroke requirements to 0.15 m with a peak load of 180 N; this was performed by an actuator with 667 N capacity and positional feedback provided through a linear potentiometer.

The extension and retraction of the boom is performed with a stepper motor-driven linear belt and pulley system. It is chosen over alternative options such as hydraulics for its light weight and ability to be compacted, its cleanliness, and its use of readily available standard components.

### 4.3. Base Maneuvering

With three independently steered and driven wheels, there is significant control over the pose of the ATI within the environmental constraints. The system developed and detailed within this section emulates a planar joint between the base and the surface of the tunnel, allowing for two-dimensional translation and one-dimensional rotation.

#### 4.3.1. ATI Drive Control Concept

Direct control of three independently controlled drive systems becomes complex for the user having two inputs required per wheel (direction and velocity). Therefore, a central control system was designed to simplify the user interface and reduce the possibility of numerous and redundant inputs. This concept is divided into two sections based on the anticipated motions required while inspecting the tunnel: a pure translation mode and a curve-following mode. Variations in these drive methods, such as front-wheel steering that mimics standard road passenger vehicles, are also configurable. Both of these modes of operation simplify the user interface by reducing the required user inputs to just two: a net ATI velocity and either a translation direction or a turn radius.

The pure translation scenario will be frequent in straight sections in which the ATI will move along the axis of the tunnel. It is also prevalent in the transverse motion of centering the ATI in the tunnel invert. This pure translation can be achieved through the control synchronization of each wheel’s direction and velocity, with a single universal direction and velocity input of the ATI base.

The curve-following or rotation mode addresses the scenario in which the tunnel being inspected consists of a horizontal curve with a known radius of curvature. This also provides means for the general reorientation of the ATI for alignment or general steering. The complications of following the curvature of the tunnel arise from the discrepancy between the location of each wheel in reference to the turn radius point. To effectively follow the curve and avoid wheel slippage, each wheel must be oriented tangential to its respective turn radius and rotated at a proportional velocity.

Further details of the control concept, the derivation of the ATI drive model dynamics, and the analytical relationship between the user inputs and each wheel’s speed and direction controls can be found in [22,29].

## 5. Control

**Figure 10:**
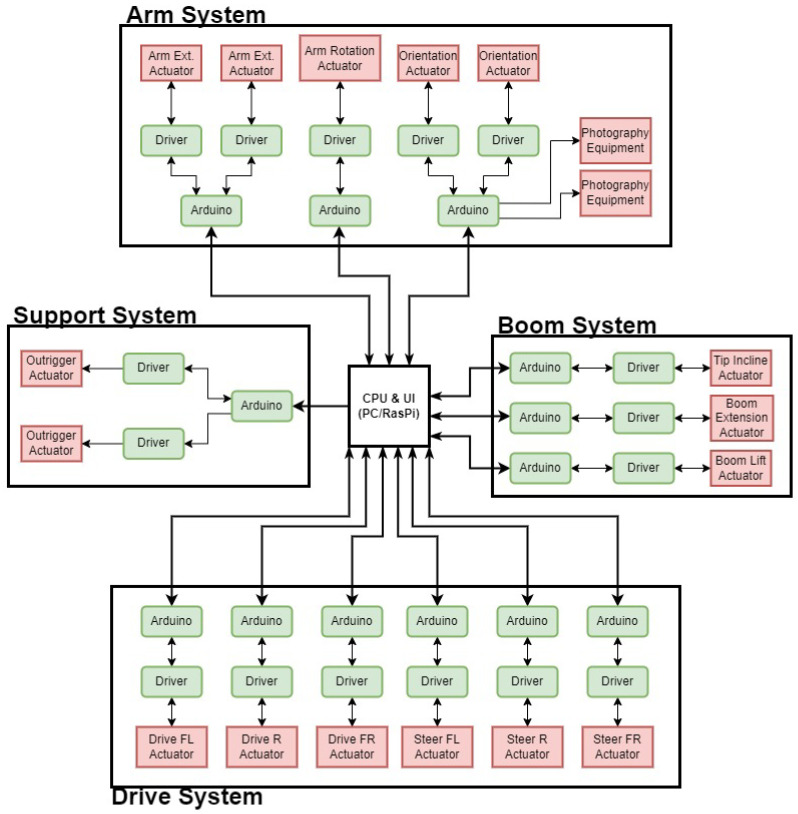
Schematic of component layout and connectivity.

The majority of the different actuators and controlling components are divided into their own subsystems with closed loop control contained within. A high-level schematic of the layout and integration of the components from the exterior actuators to the central command system and user interface is presented in Figure 10. These systems respond to high-level control inputs that are derived from the user input parameters and the overarching ATI control concepts. This section discusses the different closed-loop control techniques used for driving these systems, whether independently or synchronized with others.

A user interface was developed to connect the user with all elements of the ATI and the collection process. The interface provides multiple different methods of control for different scenarios and applications; manual control over each individual actuation, combined control of subsystems, different driving methods, and an operational scanning mode according to specified tunnel and capture parameters. This versatile setup allows the user to control the ATI from a laptop mounted to the frame, as well as the option to operate through a mobile device that can be remotely connected to the onboard Raspberry Pi.

Effective system control heavily relies on the quality of the system data or analytical models that are available. The model identification of each system was conducted experimentally by analyzing open-loop data to create empirically based analytical models of each system. As the open-loop responses did not show significant oscillatory behavior, first-order approximations were made from these data to estimate open-loop characteristics (such as time constants and gains) and formulate accurate models of each system. Simulations of these models in response to the same inputs from the experiments were conducted to determine their accuracy; MATLAB^®^ [30] was the fundamental software used for this analysis. Closed-loop transfer functions for PI control and open-loop transfer function dynamic matrices for model predictive control (MPC) were then developed and tested in simulation to evaluate performance and tune if needed before physical implementation.

The demands of all the ATI actuations vary significantly; some require velocity control, some positional control, and others a combination of both. Many of the actuations are performed at low speeds and only experience small accelerations, resulting in gradual transients in the dynamic responses. Due to this, actuations that have low peak speeds, experience small accelerations, and require only control of the end position, perform effectively using a simple ON/OFF control strategy. Aspects that require additional control of velocity throughout the entire motion utilize this ON/OFF strategy with an additional internal feedback loop, including a PI controller. Further details of these control schemes can be found in [22].

### 5.1. Arm Radius Synchronization

Synchronizing the extension of the radial arm actuators is key for maintaining the equilibrium of the arm and avoiding a significant imbalanced load scenario. A relatively small extension discrepancy between the two actuators will create a significant moment on the arm rotation joint. This could impact the arm’s ability to effectively rotate or hold its current position if the moment imposed on the rotation joint is excessive. To avoid this, the extension position of the actuators must be synchronized. Although the load carried by each of the actuators is the same, the angle at which the arm is positioned alters the driving force required to extend or retract the actuators. This, in combination with the internal resistances of the actuators and the translating assembly, could result in discrepancies between the speed of each actuator and the ultimately differing motion profiles on the way to the position setpoint. Therefore, in addition to matching the end setpoint position, the velocity throughout the extension action must be synchronized for the two translational systems as well.

**Figure 11:**
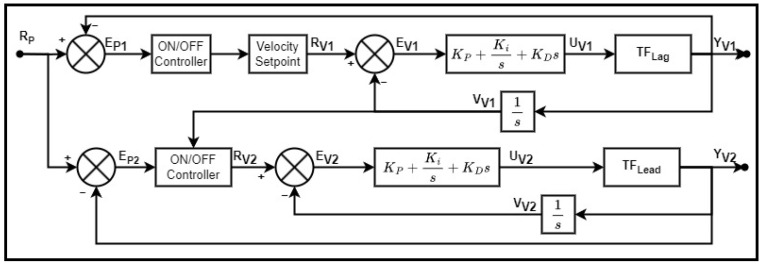
Schematic diagram for synchronized position and velocity control of the radial arm extensions.

The velocity control is formulated as a master–follower system. The corresponding master and follower system changes depending on the instantaneous relative position of each; the translational system that is lagging behind becomes the master so that the leading system can reduce its speed to match the other. The positional control is simply completed with an ON/OFF controller for each system with a single setpoint. This works by allowing the respective velocity control of the system to function until the position reaches the setpoint within a predefined tolerance. See Figure 11 for a diagram of this control scheme. Here, subscript Lag or Lead indicates which system is lagging or leading relative to the other.

**Figure 12:**
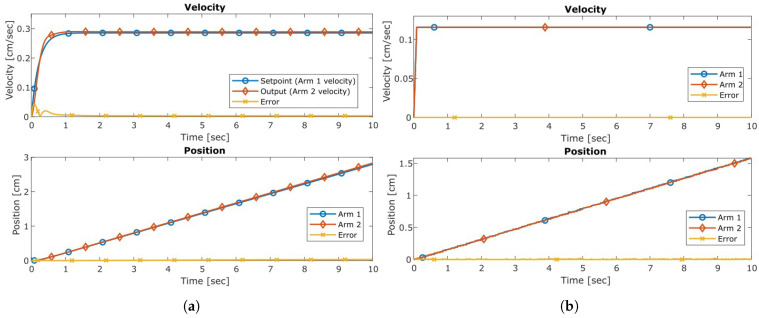
Closed-loop result comparison between the analytical and physical synchronized arm extension system. (**a**) Analytical Model. (**b**) Physical System.

**Figure 13:**
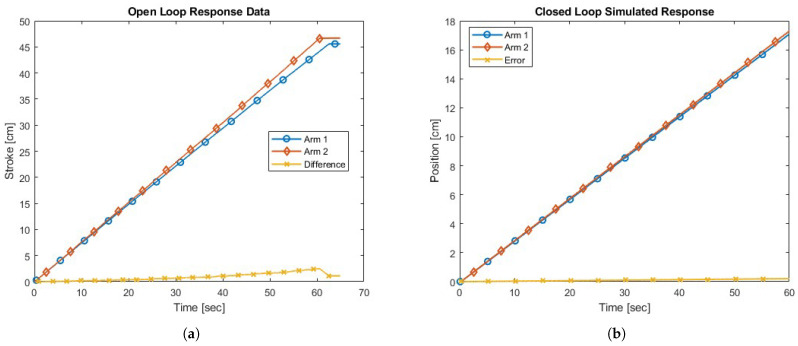
Closed-loop analytical result compared to open-loop physical response. (**a**) Open Loop: Physical System. (**b**) Closed Loop: Analytical Model.

Analytical models were created from the open-loop responses and simulated to validate their accuracy; these closely resembled the actual responses through both the transient and steady-state phases. This process identified a peak extension difference between the arms of 2.49 cm (5.7%) and a steady state velocity difference of 0.041 cm/s (5.6%). These models serve as the foundation for the control development of a PI controller, chosen for the improved steady-state response and simplistic implementation. Closed-loop specifications of a 0.5 s settling time and an overshoot of 1% defined the parameters to determine the controller gains. A demonstration of the closed control operation of the synchronized arm system in response to a setpoint of 0.28 cm/s as an analytical system and a physical experiment are shown in Figure 12. The closed-loop feedback control synchronized the system responses throughout the extension, maintaining a steady state error of 0.0035 cm/s (1.2%). Figure 13 shows the effect of the closed-loop control compared to the open-loop response. This strategy in Figure 11 significantly reduces the discrepancy between the actuator’s position throughout and ensures a more balanced arm.

### 5.2. Drive Controls

**Figure 14:**
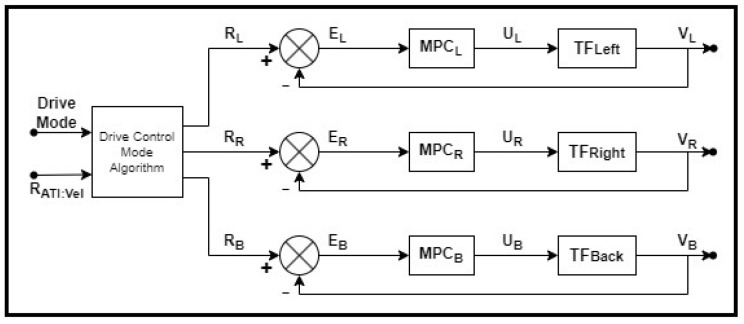
Schematic diagram for drive system controlling wheel velocities.

The control of the ATI’s drive system requires three inputs from the operator: desired drive mode, velocity of the ATI, and either direction of the ATI or the radius of curvature to be followed by the ATI, depending on the drive mode desired. The drive control mode algorithm described in Section 4.3.1 automatically determines the individual setpoints for each drive and steer motor system based on the higher-level user inputs. Subsequently, each wheel system has an independent closed-loop MPC system to follow the velocity setpoint. Figure 14 shows the schematic of this system for controlling the wheel velocities. The control of the wheel steer motors is organized in a similar configuration, albeit utilizing PI control in place of the MPC.

An open-loop response to a step input was used to construct a dynamic matrix to begin the implementation of MPC. Iterating through simulations while varying the control horizon allowed an optimal control horizon to be determined through a visual comparison. These data clearly showed that an increased control horizon experiences higher initial overshoot as a trade-off of reaching the setpoint more quickly. Conversely, the lower horizons have reduced overshoot and a slower response time. For the application of drive velocity control, limiting undesired overshoot is important for safe control and operation, as well as ensuring the maximum linear advance is maintained. The additional tuning of the other MPC parameters further improves the response, creating a smoother transient to steady-state transition and limiting the volatility of the response. Iterations of simulations while varying these parameters led to the identification of an improved and close-to-optimal set of parameters for driving these wheels. The optimal control horizon was determined to be four. A control move suppression factor of 0.2 was added, and α was chosen to be 0.8 (a factor that smoothens step changes on setpoints).

**Figure 15:**
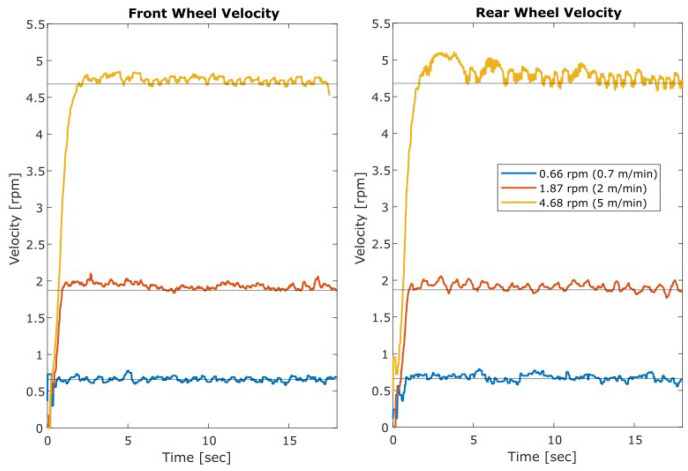
Closed loop experimental MPC response with tuned parameters.

Following satisfactory close-loop control simulations, experimental tests were conducted at various velocities to demonstrate effectiveness. Figure 15 demonstrates the response of the front wheel and the rear wheel while following different velocity profiles. A one-dimensional median filter was applied to reduce noise from these tests. The noise and volatility of the response increases proportionally with the output velocity, maintaining a relatively consistent signal-to-noise ratio. This plot shows that the control technique performed effectively over the full velocity range following the set speed with minimal overshoot or significant error in most of the test. Additional information and visual representations of the related data can be found in [22].

## 6. Photogrammetric Image Network

### 6.1. Image Sequencing

Typical methods of collecting images for photogrammetric analysis, whether in tunnels or other general areas, is a uniform grid-wise approach. The previous approach by BE, as described in Section 2.1, navigates a single camera through the cylindrical grid in a stationary and incremental sequence to create this grid. This incremental approach of stopping at each lengthwise station and capture angle during the rotation is inefficient, considering the time spent accelerating, decelerating and allowing the camera arm to settle.

**Figure 16:**
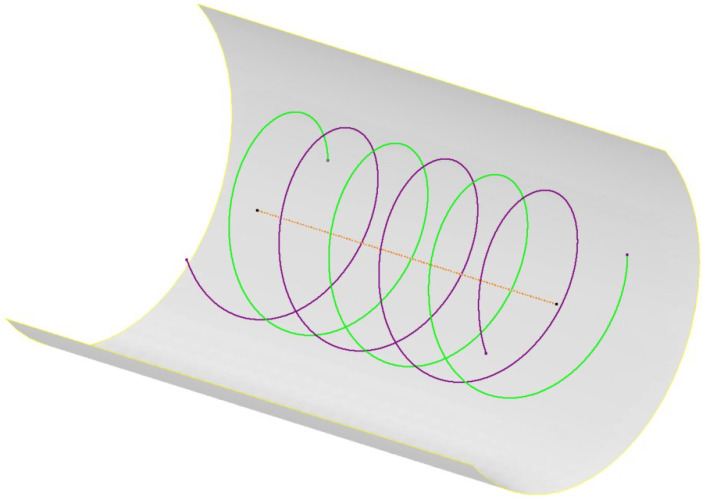
Continuous image sequence camera paths; purple and green colors differentiate each camera.

The most obvious way to improve this method is to avoid the need for stopping at each capture angle and station. This means capturing images while the camera is in motion, which imposes additional constraints not present when the camera is stationary; these are detailed in Section 6.3. Capturing images while in motion includes continuous capture and rotation while moving along the length of the tunnel; this completely alters the resulting camera path. The continuous capture results in a spiral-like sequence, as shown in Figure 16. The delays contributing to the time required to complete one stage (rotation and linear advance) are significantly reduced in this method as it becomes continuous throughout. The linear advance time is matched to the rotation time to ensure that the cameras are at the same clock location when the correct linear advance is made.

The length of the ATI arm and therefore the radius of the camera path set the distance between the camera and the surface. This parameter is a function of the desired ground sampling distance (GSD), the tunnel diameter, and the camera’s focal length. These parameters also alter the surface coverage captured within each image, subsequently changing the possible advance between images. However, adjusting the camera parameters for more optimal or alternative properties was not part of this work, and a predefined distance between the camera and the surface was set to 2 m to provide the target GSD with the predetermined equipment.

### 6.2. Image Density

The image density (represented by κ) has been defined as the number of images that capture a single point and is critical for ensuring that the quality of the resulting photogrammetric model is maintained. For an image network of a single orientation and a uniform image grid, the average image density is calculated based on the linear and rotational advances between successive images in the respective directions, as shown in Equation (16). This can alternatively be related to the image overlap (OL) as the complementary percentage to the image advance.

(16) $$\kappa =\frac{100}{\%Adv_{Lin}}\times \frac{100}{\%Adv_{Rot}}=\frac{100}{100-\%OL_{Lin}}\times \frac{100}{100-\%OL_{Rot}}$$

**Figure 17:**
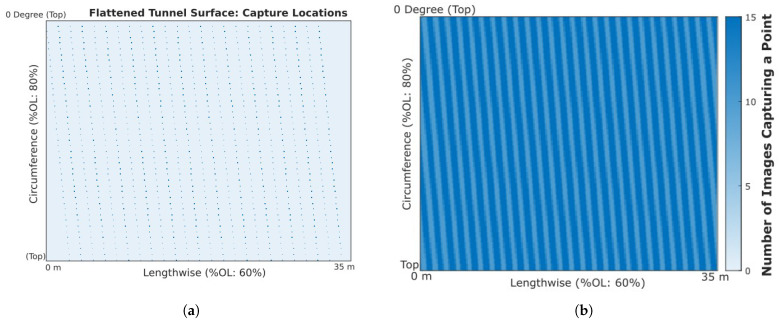
Sample scan details projected to a flattened tunnel surface. (**a**) Camera capture locations. (**b**) Image density heat map.

The simple linear grid that a piece-wise sequence creates decouples the linear and rotational OL of the images. Modifying the image capture sequence to the continuous spiral alters the resulting image grid network, resulting in a skewed grid around the tunnel. The grid that results from the continuous image sequence outlined above resembles that with the center of captured images, as displayed in Figure 17a, which shows the result for an sample image network of a 35 m long section of a 7.5 m diameter tunnel being captured with a rotational and linear OL of 80% and 60%, respectively.

Although the layout of the resulting grid is altered, the relationship between the defined image OL and the resulting image density is maintained. Figure 17b displays a heat map of the resulting image density for the same example image network displayed above; the figure shows the full frame of the tunnel surface over the sample range, with further details of the image density pattern through an extracted section of consistent capture. Extracting this section allows for the image density to be explored, identifying the minimum, maximum, and average density values. Visualizing the outcome in this form helps to identify patterns of fluctuating density or pockets of outlying densities, which may arise from the overlaps or orientation variations.

A prominent waveform pattern in the direction of motion appears in the resulting heat map as a symptom of the overlaps applied due to the linear advance (40%) not being an even factor of the lengthwise dimension of an image. This is not prevalent in the rotational direction as the advance (20%) evenly captures the rotational dimension of an image. Varying the OL in each direction can alter the possible collection speed and impact the photogrammetric analysis. Analyzing and comparing data from these image density heat maps can be used for identifying the ideal OL combination and orientation sequence for a particular tunnel.

### 6.3. Capture Rate Limitations

#### 6.3.1. Motion Blur Limitation

The motion of the camera during continuous capture creates the risk of induced blur in the images. The motion of the camera has the same effect as the motion of an object within the camera’s view. There is an allowable amount of motion that can be observed without blur being seen in the image. The motion is in terms of the number of pixels moved while the shutter is open and the flash is active. Transforming this relationship into physical terms to be related to the tunnel is a function of the GSD and the active shutter or flash speed. The faster of the two is the image exposure rate, $\nu_{Exposure}$, as shown in Equation (17).

(17) $$V_{Blur}=\frac{GSD*\delta_{Pixel}}{\nu_{Exposure}}$$

The GSD refers to the size of a pixel at the surface [mm/pixel], the $\nu_{Exposure}$ is listed in fractions of a second of exposure [seconds/exposure], and $V_{Blur}$ is the resulting velocity limit in mm/second. The parameter $\delta_{Pixel}$ is the proportion of a pixel allowed to move during exposure to avoid blur [pixels/exposure]; as a rule of thumb, this is half of the GSD [31]. This velocity relates to the velocity of the image on the surface itself and therefore, when compared to the motion speed of the ATI system, results in the magnitude of the combination of the ATI’s linear drive velocity and tangential velocity of the rotational arm translated to the tunnel surface, as defined in Equation (18). The parameter $\nu_{ATI}$ defines the linear velocity of the ATI system, $\omega_{AR}$ is the rotational velocity of the ATI arm, $r_{T}$ is the radius of the tunnel, and $V_{IMG}$ is the resulting magnitude of the net image velocity as projected to the surface.

(18) $$V_{IMG}=\sqrt{\nu_{ATI}^{2}+{\left(\omega_{AR}*r_{T}\right)}^{2}}<V_{Blur}$$

Equation (19) defines the ratio between linear velocity and rotational velocity translated to surface $\nu_{Ratio}$, which relates the linear displacement of the ATI when the arm makes one complete rotation ($\delta_{Lin/Rot}$) with the projected circumferential surface displacement of the images throughout that rotation ($2\pi r_{T}$). Maintaining this relationship is critical to ensure the spiral camera path and required OL between successive images are obtained.

(19) $$\nu_{Ratio}=\delta_{Ratio}=\frac{2\pi r_{T}}{\delta_{Lin/Rot}}$$

#### 6.3.2. Flash Refresh Rate

In addition to the direct limitations on the physical motion speed of the cameras, constraints are placed on the image capture rate due to aspects of the photography equipment. As each camera has a set of two flashes that operate at close to full power to provide sufficient lighting for the images, image capture frequency significantly affects the power consumption and the rate at which the flash can take successive images: the flash refresh rate (FRR). As the frequency of image capture increases, the risk of overheating increases as well. If a fast flash rate is sustained for an extended period, the FRR will be automatically reduced or completely halted for a period to cool-down. The flashes used were the AD400Pro from GODOX^©^ Photo Equipment Co. The product manual documentation lists the number of flash actions that will activate over-temperature protection for a particular output power; at an output power of $1/4^{th}$ of full power (originally estimated as the required flash power for capture in Grand Falls), this is 200 flashes [32]. However, the rate that is deemed “fast succession” is not specifically defined in the documentation. To identify an acceptable capture rate that would allow the flashes to continuously operate without any deviance in the flash performance, experimental tests were conducted. This conservatively concluded that a flash rate of five seconds would ensure continuous and reliable flash operation.

The $FRR_{max}$ is the maximum allowable flash refresh rate, as detailed below. The resulting capture rate subsequently imposes a constraint on the overall arm rotation, which varies with the number of images required per rotation or the resultant angle between successive images ($\phi_{inc}$) as defined in Equation (20). The resulting net velocity limit related to the camera motion ($V_{FRR}$) is defined in Equation (21).

(20) $$\omega_{FRR}=\frac{\phi_{inc}}{FRR}$$

(21) $$V_{FRR}=\sqrt{{\left(\frac{\omega_{FRR}}{\nu_{ratio}}\right)}^{2}+{\left(\omega_{FRR}*r_{T}\right)}^{2}}$$

Consideration of both of these limitations is key to ensuring effective capture, but only one will be the critical factor at a given time (Equation (22)):

(22) $$ICR_{Limit}=min\left(V_{Blur},V_{FRR}\right)$$

## 7. Results and Discussions: Scanning of the Grand Falls Intake Tunnel

The powerhouse at the Grand Falls in New Brunswick houses four Francis turbines with a total capacity of 66 MW. Water is diverted to this station from the Saint John River through an intake tunnel running under the town center. This tunnel is approximately 920 m in length, consisting primarily of an 822 m long concrete lined section.

Visual walkthrough inspections have been the primary method of inspection for decades and continue to be conducted to identify current defects and areas for caution or immediate repair. The first photogrammetric scan of this tunnel was completed by BE in 2017 [1]. This scan captured about 5000 images over 7 days using the TIA system and methodology, as described in Section 2.1. These images were captured at a visual resolution of 1.3 mm with 80% rotational and 60% longitudinal OL [1]. The details and experience of the updated scan completed with the ATI is discussed within this section.

### 7.1. Acquisition Process Discussion

The total image acquisition process spanned just over four working days, each being a scheduled 10 h work day (7:00 a.m.–5:00 p.m.); however, the general tailboard meetings, safety discussions, and uncontrollable time spent waiting for entrance to the tunnel allowed for approximately seven working hours in the tunnel per day, which were broken into two sessions. Actual scanning time was further reduced due to the daily set up and tear down of the camera equipment and the preparation of the ATI.

**Figure 18:**
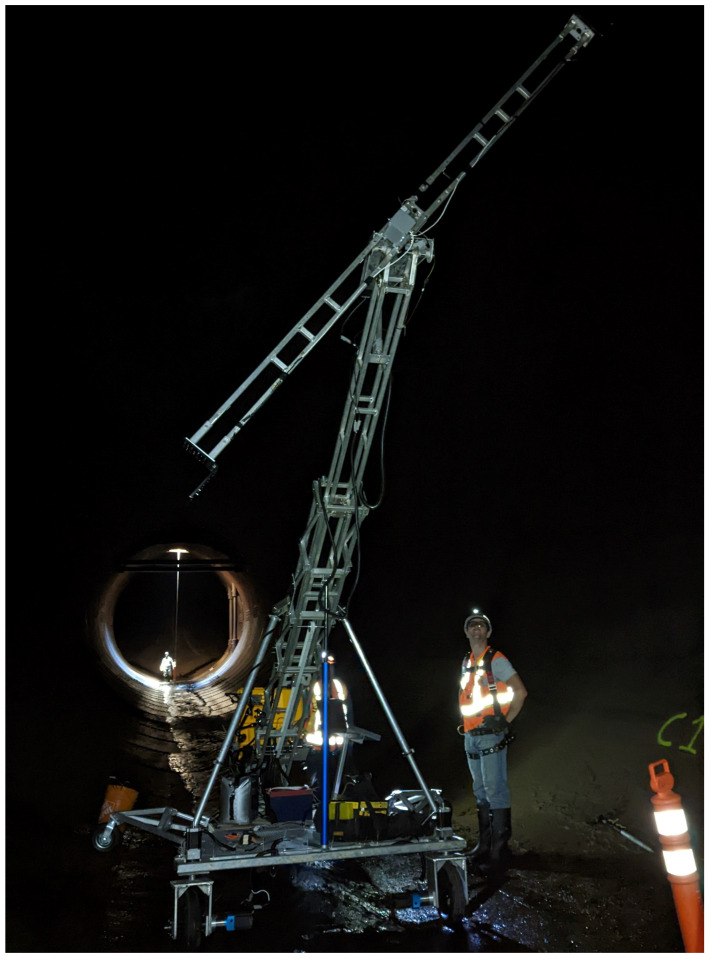
ATI assembled upstream from equipment hoist location.

The ability to bring and set up equipment in the tunnel through the main access port, which involved transporting through a small porthole and the use of a rope hoist, simplified the process compared to the special treatment that was required to transport TIA into the tunnel in 2017, validating a benefit of the new modular design of the ATI. Figure 18 shows the ATI being assembled, with reference of the hoist location in the background.

As this was the first time setting up and operating within the actual tunnel environment, its behavior on the muddy, slippery, and rough tunnel surface was thoroughly tested before confidently beginning the actual scanning process. Complete scanning began and proceeded steadily for approximately four and a half working days. Much of this process was smooth, and minimal interjections or interrupts were experienced; however, some locations within the tunnel required additional attention. A number of locations along the tunnel had hose-sized sprays coming from the top or sides of the tunnel, which were to be avoided if possible to protect the camera equipment. In many cases, risk of water contact with photography equipment could be mitigated by using extended umbrellas to divert the sprays away, while other cases required alteration to the spiral scan sequence to ensure as many images were captured around these locations as possible with sufficient OL, while still mitigating water contact with the cameras.

In addition to the presence of water, rough terrain at the invert of the tunnel required cautious attention throughout, as there were a collection of defects, grooves and potholes of up to ten centimeters deep that a wheel could fall into. For the most part, these were avoided if noticed; however, it was not uncommon for a wheel to slip into one of these, which may have caused some slight jerking or skewing of the ATI but did not cause a significant interference. It was slightly offset from the center of the invert and was rectangular in shape, measuring approximately 2.5 m long, 0.75 m wide, and over 0.5 m deep. Scenarios like this are a prime example of the extreme complexity of fully automating the motion of the ATI in these types of environments.

### 7.2. Results and Comparisons

Since the first scan in 2017, the abilities of the photogrammetric analysis performed by BE have steadily improved from the former methods and technologies, which now allow for images to be captured at a resolution of 0.4 mm for these tunnels. This requires a camera–surface distance of 2 m. This camera offset, combined with the desired 80%/60% image OL and other properties of the photography system, resulted in the choice of 52 images per rotation. The ICR, as defined in Section 6.3, was set to 5 s due to the most critical limitation imposed by the flash refresh time. Collecting this number of images at that rate sets the rotational speed to 1.34 degrees/second and results in a total rotation time of 4.5 min.

Using a dual camera system allows for twice the linear advance to be made during one complete rotation, as same-angle images will be captured by opposing cameras every half rotation. Synchronizing the linear advance (40%) with the timing of the rotation sets the linear speed of the ATI to 0.67 m/min. At this rate, the 822 m section of the tunnel was estimated to require approximately 23 h of scanning time, assuming perfect efficiency; this turned out to be a reasonably accurate estimation, considering the delays and interference with the ability to scan that slowed the overall process at times. A similar estimation for the ATI to perform a scan of similar quality and resolution to the scan conducted in 2017 shows a total required capture time of 7 h. While this assumes ideal efficiency and does not include the time for set up and interruption, a drastic improvement in automation, control, and image capture over the approach taken in 2017 is evident.

**Figure 19:**
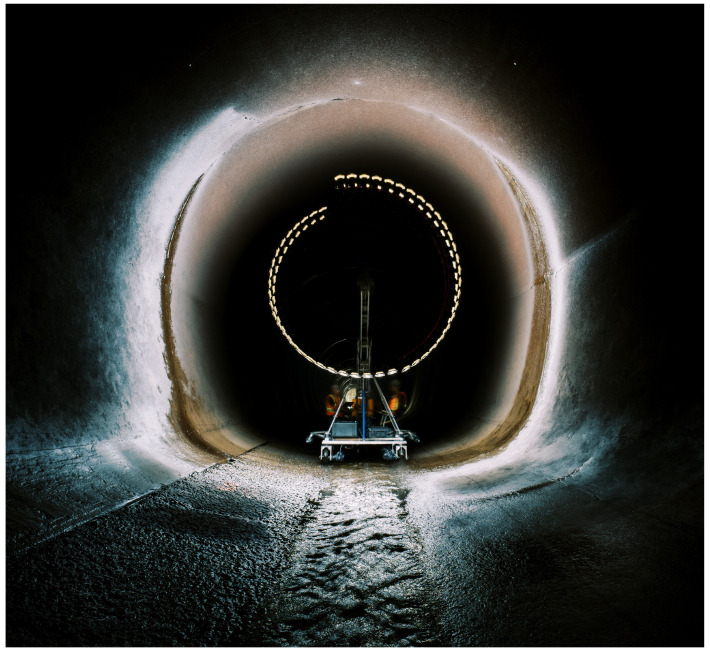
Long exposure photo of the ATI scanning the tunnel.

Table 4 summarizes the key differences and improvements between the scan performed by the ATI and the previous scan in 2017. At the end of the scan, a total of approximately 28,000 images were captured throughout the tunnel, drastically higher than the number of images captured previously. While the sequence was completed with the same linear and rotational OL, the increased resolution and smaller camera–surface distance provide the explanation for the significant increase in images required to capture the same tunnel. Collecting such a large number of images at a higher resolution and in a shorter time period demonstrates the advantages of the new ATI system and the continuous spiral sequence developed. Figure 19 shows a long exposure photo of the ATI during the scan of the tunnel; this image records a 180 degree rotation of each camera.

## 8. Conclusions

### 8.1. Conclusions

As discussed, a semi-automated camera positioning system, referred to as the ATI, was designed and developed to address a set of limitations identified in the data acquisition process of photogrammetric inspections in hydroelectric tunnels. ATI utilizes a variety of electromechanical components and advanced predictive control techniques to drive and automate elements of the image collection process. The ATI design is capable of automatically adapting to tunnel diameters between 4.5 m and 9 m and maintaining consistent image capture parameters at any variation. Modification of the camera-to-surface distance can be used to vary the image resolution and alter the total scan procedure. The ATI allows for complete driven mobility, including pure translation or rotation for effective reorientation and alignment within the tunnel environment.

The model quality constraints, photography equipment properties, and photogrammetric parameters formed the basis for the required image capture parameters throughout the tunnel. A unique spiral image network was developed that allows the full potential of the ATI to be utilized by facilitating continuous image capture while rotating and driving along the tunnel. This sequence eliminated any idle time that creates inefficiencies in the process that had been observed in the previous, more incremental capture sequences. The rate of capture limits were quantified based on the motion limit to avoid blur in the images, the refresh rate of the flashes, and the time required to change orientation, all of which vary depending on tunnel and photogrammetric parameters. This analysis optimizes the rate of capture of the continuous approach and promotes efficiency in the collection process. In the process of the development and implementation of the ATI, advanced closed-loop control techniques, including various PI-based and model predictive control schemes, have been formulated and assessed in a unique and challenging environment on this large scale robotic system.

### 8.2. Future Work

While the direct goal of this work is to make significant improvements to a semi-automated system, this work can serve as a foundational element for future research in the pursuit of an autonomous ATI.

- Further research into localization within GNSS-denied, dark, featureless environments should be conducted to improve the accuracy and consistency of real-time localization within a tunnel to identify the complete pose of a vehicle in these challenging environments. This area of research is foundational for other automation-related applications.
- Maintaining alignment of the ATI while moving throughout the tunnel required frequent steering adjustment, and although fully automating this was outside of this project’s scope, it stands as an aspect for significantly reducing the efforts and attention required of the operator. The self-navigation of a three-wheeled vehicle in a generally cylindrical tunnel with the goal of maintaining centricity, alignment, and velocity would be a significant advancement in this aspect. This would include maneuvering in dynamic environments, and involving object avoidance methods would be a key element to this.
- The control systems for each portion of the ATI were created as different configurations of SISO systems. Further research into the system identification of MIMO systems would formulate a more optimal control scheme, particularly in the case of the model predictive control of the drive system.

## 9. Patents

Elements of this work are included in currently pursued patents.

## Figures and Tables

**Table 1:** Simulation Summary.

Action	ROM	${\mathrm{Load}}_{\mathrm{Peak}}$
Boom Inclination	0.8 m	1500 N
Boom Extension	1.3 m	460 N
Tip Inclination	0.15 m	180 N
Arm Extension	0.45 m	145 N
Orientation Change	90${}^{\circ }$	5 Nm
Arm Rotation	180${}^{\circ }$	15 Nm

**Table 2:** Modified Denavit-Hartenberg Parameters of the ATI.

Link	$\alpha_{i-1}$	$a_{i-1}$	$d_{i}$	$\theta_{i}$
1	0	0	0	$\theta_{B}^{*}$
2	π/2	$l_{2}$	$D_{B}^{*}$	0
3	−π/2	0	0	$\theta_{T}^{*}$
4	0	$l_{4}$	$d_{4}$	0

**Table 3:** Parameter definitions for driving actuators of the lift and tip joints.

Lift		Tip	
**Label**	**Value**	**Label**	**Value**
$\alpha_{L}$	BC	$\alpha_{T}$	EG
$\beta_{L}$	BD	$\beta_{T}$	EF
$\gamma_{L}$	AB	$\gamma_{T}$	EH
$\lambda_{L}$	BJ	$\lambda_{T}$	EJ
$\phi_{L}$	AC	$\phi_{T}$	HG
$\rho_{L}$	DJ	$\rho_{T}$	FJ
$D_{L}$	DC	$D_{T}$	GF

**Table 4:** Grand Falls Tunnel Scan Comparison.

	2017	2022
Overlap	80%/60%	80%/60%
Number of Images	∼5000	∼28,000
GSD	1.3 mm	0.4 mm
Camera	Canon 6D	Sony α7R III
Camera Resolution	20.2 MP	42.4 MP
Lens Focal Length	20 mm	21 mm
Camera-Surface Distance	5 m	2 m
Scan Time	7 days	4.5 days

## Data Availability

The data presented in this study are available on request from the corresponding author. No new data were created or analyzed in this study. Data sharing is not applicable to this article.

## Abbreviations

ATI	Automated Tunnel Inspector
BE	Bradley Engineering Ltd.
FRR	Flash Refresh Rate
Grand Falls	Grand Falls Generating Station
GSD	Ground Sampling Distance
Mactaquac	Mactaquac Generating Station
MAV	Micro Aerial Vehicle
MIMO	Multi-Input-Multi-Output
MPC	Model Predictive Control
mDH	modified-Denavit-Hartenberg
NB Power	New Brunswick Power Corporation
OL	Overlap
ROM	Range of Motion
SISO	Single-Input-Single-Output
TIA	Tunnel Inspection Assistant
